# Preparation of Agarose Fluorescent Hydrogel Inserted by POSS and Its Application for the Identification and Adsorption of Fe^3+^

**DOI:** 10.3390/gels7040173

**Published:** 2021-10-18

**Authors:** Zhengquan Fu, Ming Li, Yuanhang Li, Zhiyuan Zhang, Di Wang, Chengyu Wang, Jian Li

**Affiliations:** 1 Key Laboratory of Bio-Based Material Science and Technology (Ministry of Education), Northeast Forestry University, Harbin 150040, China; fzq1999fzq@163.com (Z.F.); alexlavie@outlook.com (M.L.); lyh@nefu.edu.cn (Y.L.); blairzhang96@outlook.com (Z.Z.); wangcy@nefu.edu.cn (C.W.); nefujianli@163.com (J.L.); 2 Engineering Research Center of Advanced Wooden Materials (Ministry of Education), Northeast Forestry University, Harbin 150040, China; 3 Collage of Material Science & Engineering, Northeast Forestry University, Harbin 150040, China

**Keywords:** fluorescent hydrogel, citric acid POSS, theoretical calculation, Fe^3+^ identification and adsorption

## Abstract

After entering in water, Fe^3+^ is enriched in the human body and along the food chain, causing chronic poisoning and irreversible harm to human health. In order to solve this problem, we synthesized citric acid POSS (CAP) from aminopropyl POSS (OAP) and citric acid. Then, we synthesized fluorescent hydrogels (CAP-agarose hydrogel, CAHG) with CAP and agarose. The luminescence mechanism of CAP was investigated by theoretical calculation. CAP plays a dual role in composite hydrogels: one is to give the gels good fluorescence properties and detect Fe^3+^; the second is that the surface of CAP has a large content of carbonyl and amide groups, so it can coordinate with Fe^3+^ to enhance the adsorption properties of hydrogels. The experimental results show that the lowest Fe^3+^ concentration that CAHG can detect is 5 μmol/L, and the adsorption capacity for Fe^3+^ is about 26.75 mg/g. In a certain range, the fluorescence intensity of CAHG had an exponential relation with Fe^3+^ concentration, which is expected to be applied to fluorescence sensors. Even at a lower concentration, CAHG can effectively remove Fe^3+^ from the solution. The prepared fluorescent hydrogel has great potential in the field of fluorescent probes, fluorescent sensors, and ion adsorption. Besides, CAHG can be used as photothermal material after adsorbing Fe^3+^, allowing for material recycling and reducing material waste.

## 1. Introduction

As a common metal ion, Fe^3+^ is harmless to the human body. However, after it enters in water, it is enriched in the human body and along the food chain, causing chronic poisoning and irreversible harm to human health [1,2]. Belonging to the functionalized hydrogels, fluorescent hydrogels are often used in ion detection and other fields [3,4,5,6]. Typically, fluorescent hydrogels can be obtained by using luminescent materials, such as organic fluorescent dyes [7,8], rare earth elements [9,10,11], and biomass carbon dots [12,13,14]. Alongside its new properties, fluorescent hydrogels retain the good mechanical, chemical, and biological properties of typical hydrogels [15,16]. Usually, traditional fluorescent hydrogels are based on polyacrylamide [17,18] and polyvinyl alcohol [19,20]. However, the non-biodegradability and weak biocompatibility of these synthetic polymer hydrogels limit their applications. Compared with synthetic polymers, biomass polysaccharides, such as cellulose, chitosan, and agarose, have good renewability and biocompatibility, and thus have been regarded as promising candidates for preparing high-performance hydrogel materials [21,22,23]. Fluorescent hydrogels based on agarose can not only detect metal ions, but also adsorb ions [24]. This may solve the problem of the limited applications [25,26] of traditional fluorescent hydrogels.

The sensing and detection functions of fluorescent hydrogels are mainly achieved through the introduction of light-emitting groups. However, the widely used light-emitting groups, such as organic fluorescent dyes and rare earth elements [7,8,9,10,11,20], limit the scope of application due to their non-biocompatibility. Although biomass carbon dots have good biocompatibility, most carbon dots have an aggregation-caused queuing (ACQ) effect [27,28,29], which weakens their application effect. Hence, the development of a material with good biocompatibility and luminescence in the aggregated state is required. The opposite of the ACQ effect is the aggregation-induced emission (AIE) effect [30]. The cause of the AIE effect is the obstruction of intramolecular motion. This discovery effectively solved the problem of luminescence weakening of luminescent materials in a concentrated solution or solid state [31,32]. Polyhedral oligomeric silsesquioxane (POSS) is a multifunctional organic/inorganic nanomaterial with good biocompatibility. POSS has reactive or nonreactive groups on the outside. Reactive groups can realize chemical bonding between the POSS and polymers, and nonreactive groups can improve the compatibility between the POSS and polymers [33,34]. The cage structure of POSS has a steric effect, which can give the fluorescent material an AIE effect [35]. Therefore, introducing a POSS fluorescent group with good biocompatibility into hydrogels can solve the above problems.

**Scheme 1:**
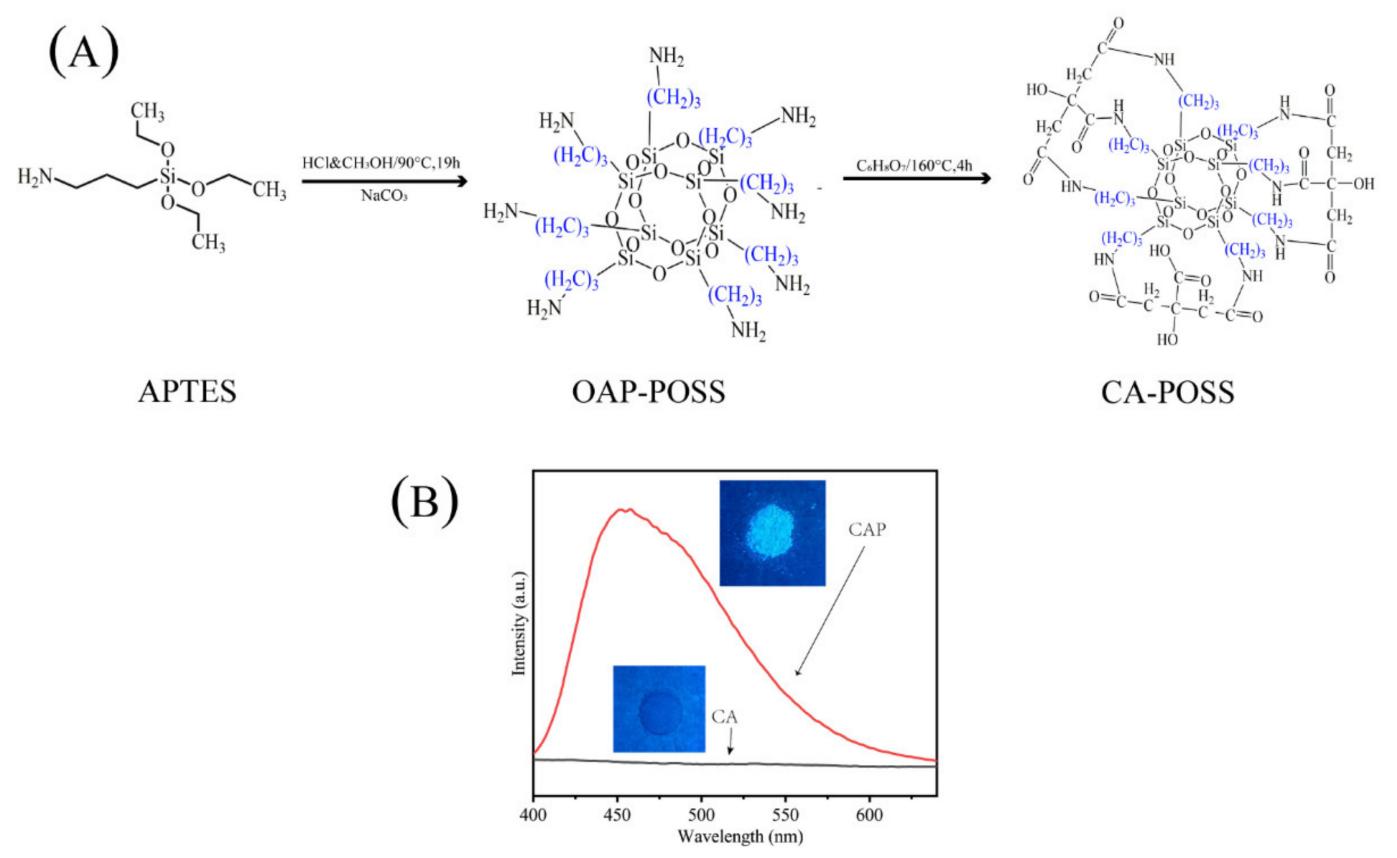
(**A**) Synthesis path of CAP (citric acid POSS); (**B**) photos of CA (citric acid) and CAP under UV (ultraviolet) lamp, and fluorescence spectrum of CA and CAP.

The aim of this study was to prepare a widely usable fluorescent hydrogel, and to detect and adsorb certain metal ions. As a natural organic acid, citric acid has many excellent properties. Therefore, it is often used to prepare fluorescent materials, and gives materials the ability to detect metal ions [36,37]. Consequently, citric acid POSS (CAP) with fluorescence properties was synthesized by citric acid and aminopropyl POSS (OAP) for the first time. Scheme 1 is the synthetic method and fluorescence properties of CAP. Its luminescence mechanism was explored by computer theoretical calculation. The experimental results show that CAP has good fluorescence properties and fluorescence-quenching behavior. Using agarose as a substrate, and by adding CAP into the agarose, a fluorescent hydrogel (CAHG) was prepared. Under the combined action of agarose and CAP, CAHG could not only be used to detect Fe^3+^ in solution, but also to adsorb Fe^3+^. Through experiments, we found that in a certain concentration range, the fluorescence intensity of CAHG had an exponential relation with the Fe^3+^ concentration, and the fitting equation was obtained. This showed that CAHG has the potential to be used as a fluorescence sensor. This study provides a potential functional biomass fluorescent hydrogel for the detection, sensing, and adsorption of Fe^3+^.

## 2. Results and Discussion

### 2.1. Structural Analysis

**Figure 1:**
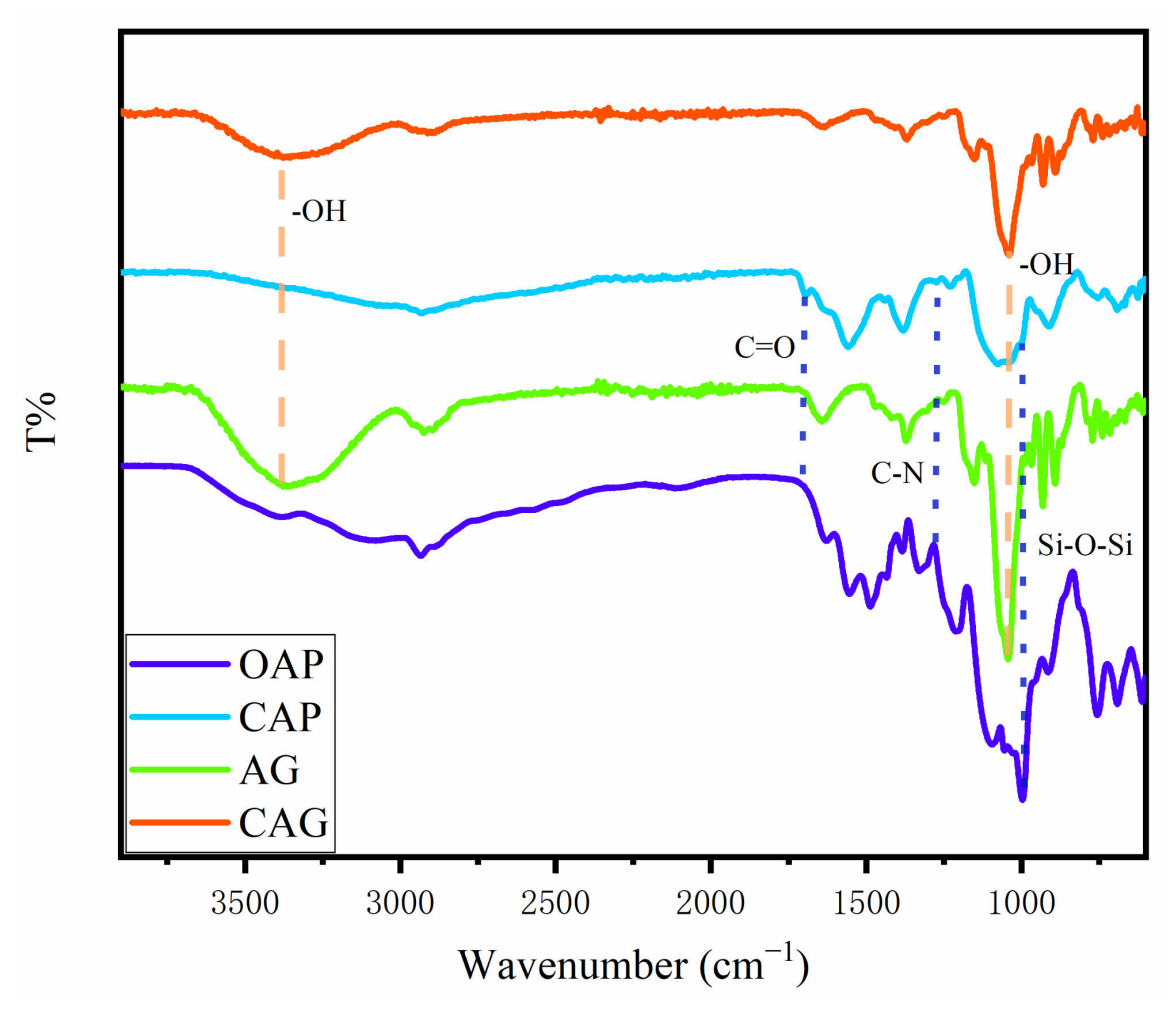
FTIR spectra for aminopropyl POSS (OAP), citric acid POSS (CAP), AG (freeze-dried AHG) and CAG (freeze-dried CAHG).

In the infrared spectrum (Figure 1), the Si-O-Si band at 1000 cm^−1^ can confirm the core structure of OAP, so it can prove the successful synthesis of POSS [38,39]. In addition, the infrared peak at 1208 cm^−1^ belongs to the C-N stretching vibration on the eight aminopropyl groups of POSS, the bands at 3385 cm^−1^ and 3095 cm^−1^ belong to the N-H stretching vibration, and the infrared peak at 1628 cm^−1^ belongs to the N-H bending vibration. After reacting with citric acid, the Si-O-Si band at 1000 cm^−1^ showed no significant changes, which means that the cage core structure remained intact. In addition, the peak at 1702 cm^−1^ belongs to the C=O stretching vibration, which also proves that the amino group in POSS reacted with the citric acid. Moreover, the shift of the C-N stretching vibration peak at 1394 cm^−1^ proved the formation of tertiary amines, which proved the formation of CAP. Figure S1 and Table S1 are the MS information of OAP and CAP, respectively, which confirmed that the OAP and CAP were synthesized successfully without impurities.

In order to characterize the hydrogel by infrared spectroscopy, we freeze-dried the agarose-hydrogel (AHG) and CAP-agarose hydrogel (CAHG), and named them AG and CAG. In the infrared spectrum (Figure 1), the absorption at 1050 cm^−1^ was the stretching vibration of the -OH on the side chain of the agarose, and the stretching vibration peak of the hydroxyl group in AG at 3374 cm^−1^. From the infrared spectrum of the CAG, it can be seen that the absorption peaks at 1050 cm^−1^ and 3374 cm^−1^ is obviously weakened, and the hydroxyl peak at 3374 cm^−1^ widens. This is due to the fact that the Si-O-Si bond contained in the composite material covers the stretching vibration of some of the original alcohols on the side chains of the agarose. Additionally, the POSS-based functional materials introduced amino groups, so that the N-H stretching vibration peak of the amino group coincides with the hydroxyl peak. We proved that the POSS-based functional material can be fully attached to the hydrogel after recombination.

### 2.2. Morphology Analysis

**Figure 2:**
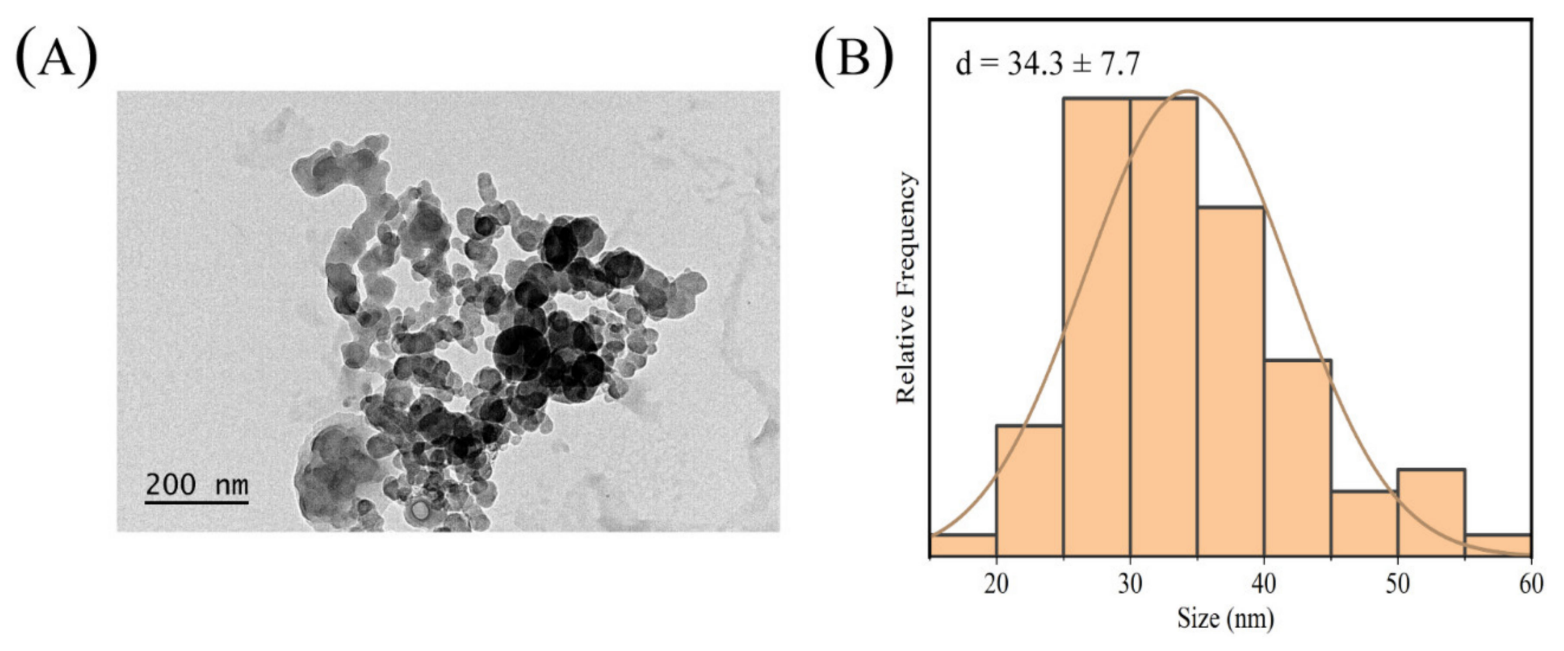

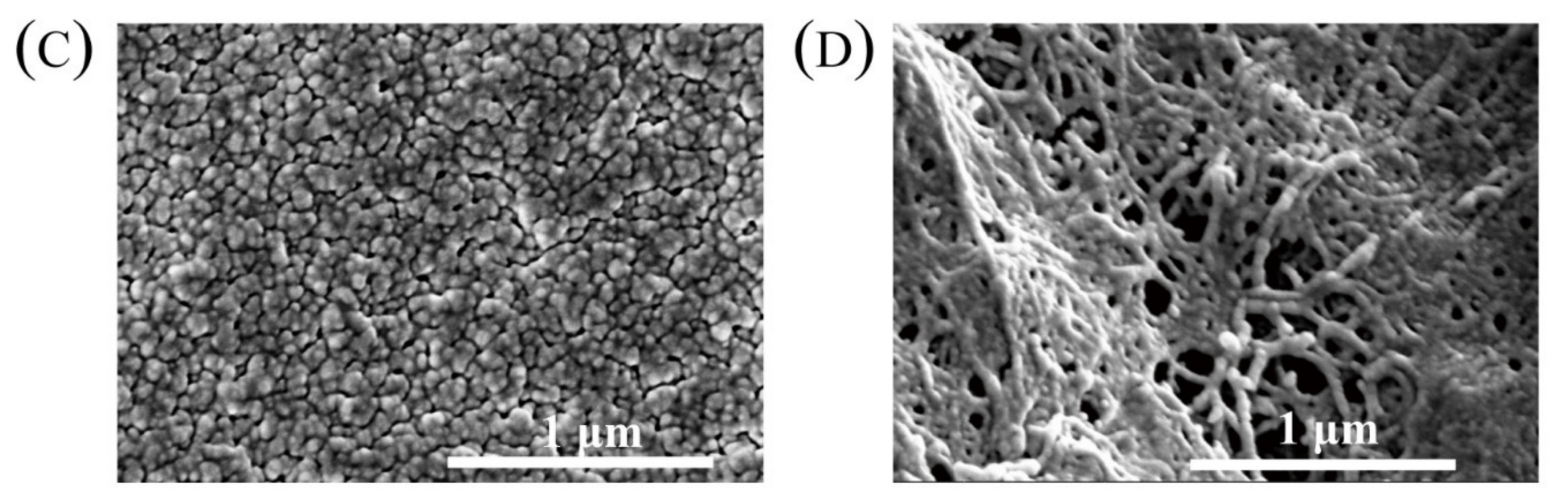
(**A**) TEM image of CAP in water and (**B**) particle size profile; SEM microscopic images of (**C**) AG and (**D**) CAG.

The morphology and structure of CAP was characterized by transmission electron microscopy (TEM). As shown in Figure 2A, the CAP imaging results show that CAP is easy to agglomerate into irregular spherical particles with an average diameter of 34.3 ± 7.7 nm and show the formation of agglomerates (Figure 2B). The change proves that CAP is easy to aggregate. As shown in Figure 2C,D, we used a scanning electron microscope (SEM) to observe the morphology of AG and CAG (freeze-dried AHG and CAHG). The microscopic SEM image of AHG shows a uniform particle distribution [40,41]. When CAP was doped in agarose, the microstructure of the agarose changed obviously, becoming fibrous and slightly relaxed compared with the original tight arrangement structure. The above results show that when CAP was added, the microstructure of the hydrogel changed significantly, and the hydrogel showed a porous structure. The reason for this phenomenon may be that some carboxyl groups on CAP reacts with the hydroxyl groups on agarose. Additionally, the unreacted CAP particles were evenly dispersed in it through intermolecular hydrogen bonds (Figure S2).

### 2.3. X-ray Photoelectron Spectrometry Analysis of CAP

**Figure 3:**
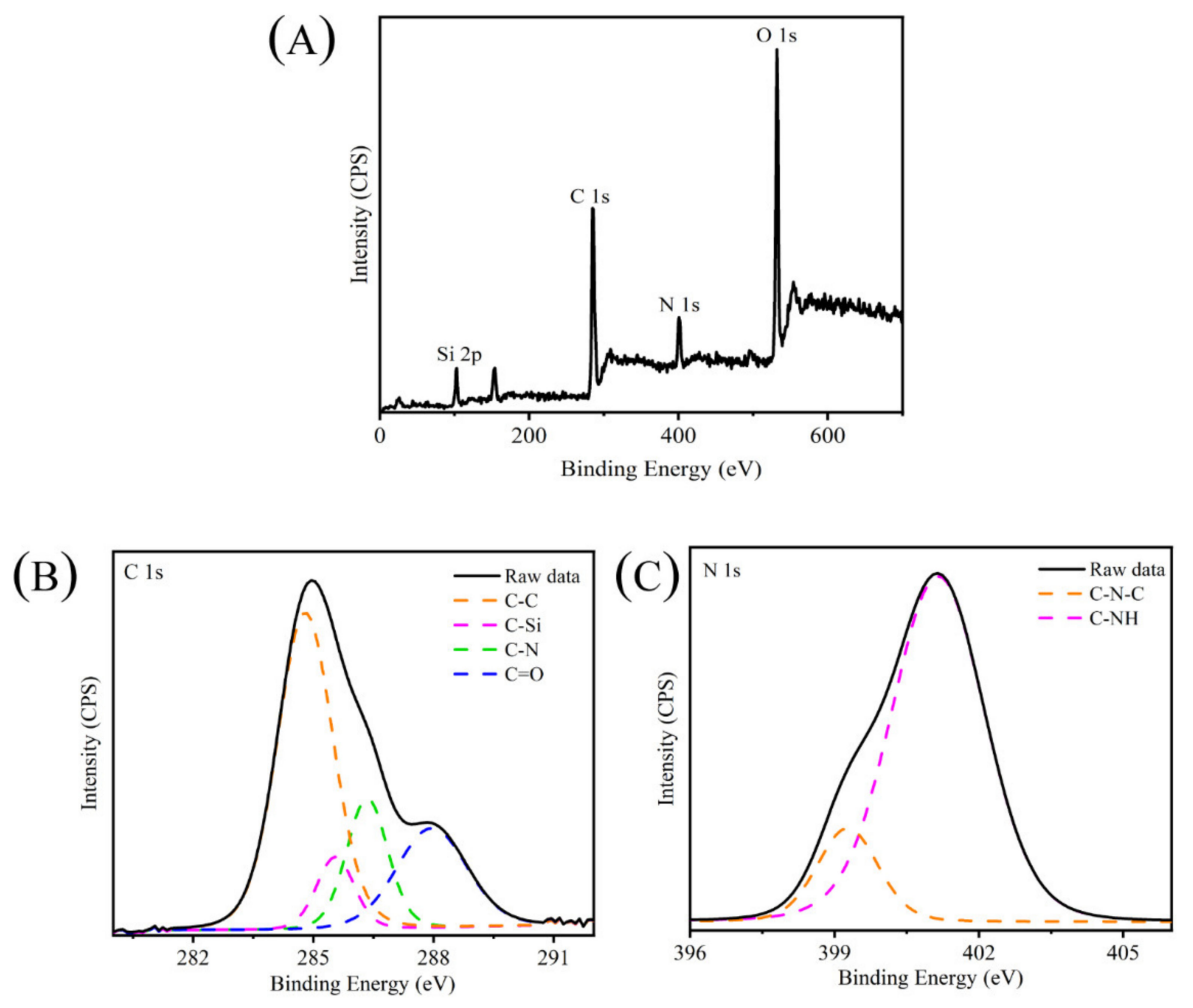

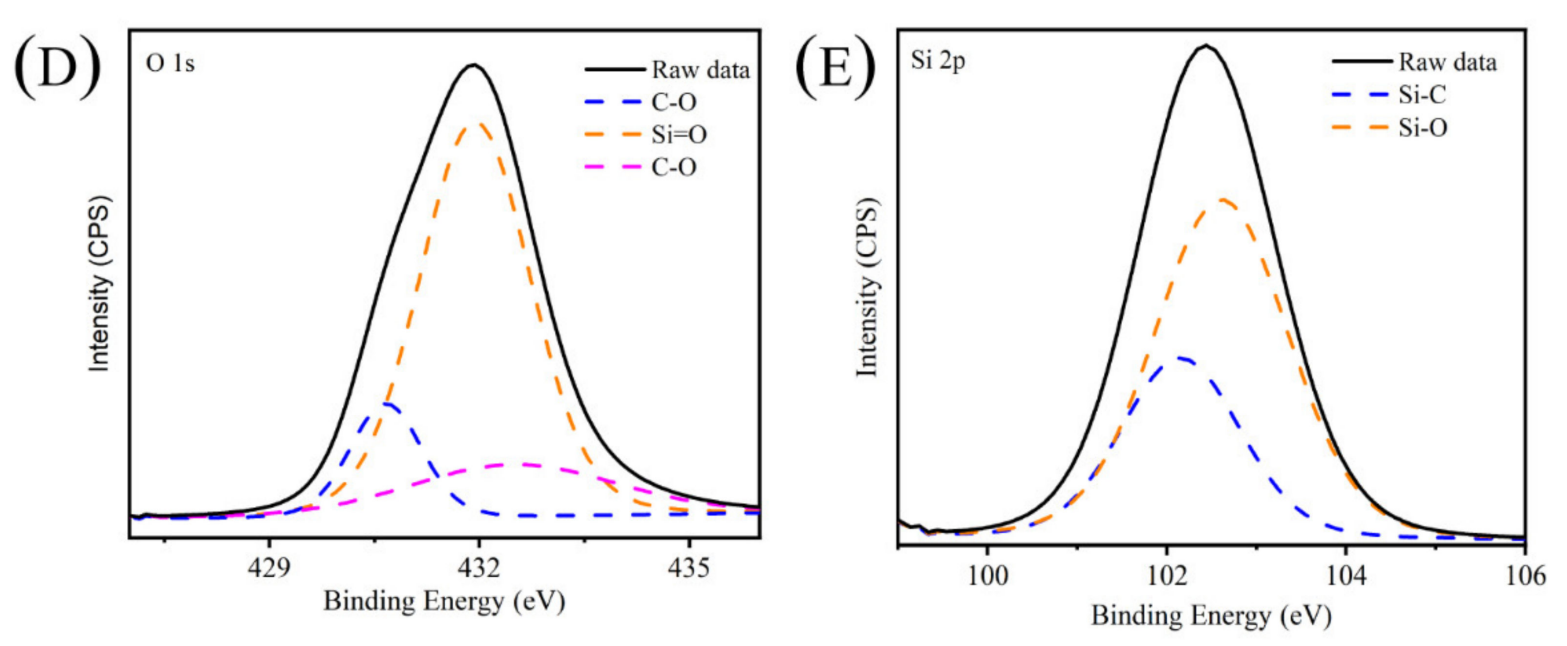
Survey XPS spectrum of CAP (**A**) and the high-resolution XPS peaks of C 1s (**B**), N 1s (**C**), O 1s (**D**), and Si 2p (**E**).

In the full XPS spectrum of CAP (Figure 3A), the binding energy signals at 102.8 EV, 284.8 EV, 400.8 EV, and 531.8 EV can be attributed to the electronic binding energies of Si 2p, C 1s, N 1s, and O 1s of CAP, respecitvely [42]. The N and Si elements are mainly derived from OAP-POSS. The presence of four elements at the same time indicated that CAP was successfully obtained by the reaction of citric acid and OAP. Figure 3B shows the existing types of carbon elements in CAP, including C-C (284.80 EV), C-Si (285.55 EV), C-N (286.34 EV), and C=O (287.95 EV). Figure 3C shows the types of nitrogen in CAP, which are C-N-C (399.27 EV) and C-NH (401.15 EV). Figure 3D shows the modes of oxygen in CAP, which are C-O (430.66 EV), C=O (431.93 EV), and Si-O (432.47 EV). Figure 3E shows the existing types of Si elements in CAP, which are Si-C (102.15 EV) and Si-O (102.61 EV).

### 2.4. Thermal Stability and Mechanical Properties of Agarose Hydrogel

**Figure 4:**
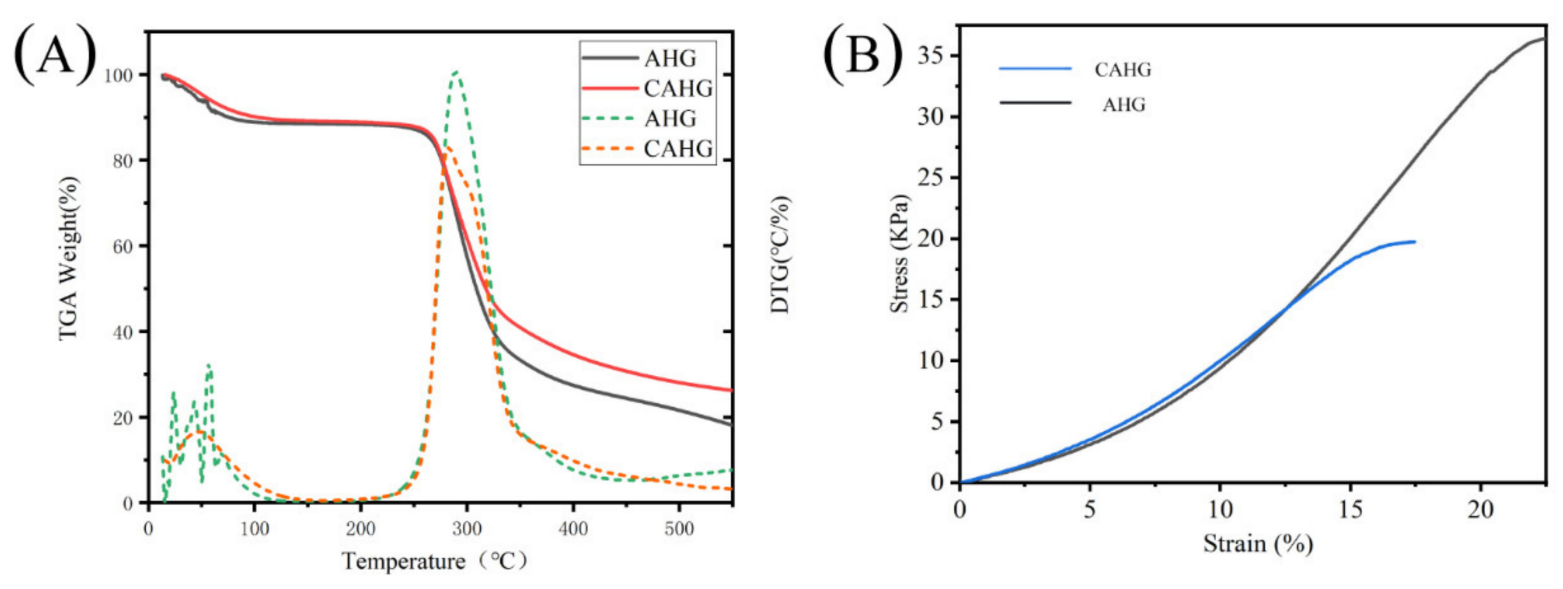
TGA (solid line) and DTG (dotted line) of AHG and CAHG (**A**); stress-strain curves of AHG and CAHG (**B**).

Figure 4A is the TGA and DTG curves of AHG and CAHG. The whole thermal decomposition process is mainly divided into two stages, as shown in Table 1. The first stage starts at 25 °C, mainly due to the presence of a small amount of bound water in AHG. The weight of AHG was almost unchanged at 125–200 °C, and the weight decreased rapidly at 240–434 °C. This was mainly due to the single structure of the agarose chain [43]. For CAHG, the weight of the composite water was reduced after evaporation at 25–143 °C. The weight of the composite hydrogel was not changed at 143–200 °C, and the weight loss at 240–471 °C was less than for AHG. This is on account of the cage-type POSS structure in CAP having better thermal resistance. With the increase in temperature, the CAP is decomposed into SiO_2_ and attached to the agarose surface. Hence, it can protect the agarose from pyrolysis and increase the residual. From the above results, we know that introducing CAP into agarose hydrogel can effectively increase the thermal stability of the agarose hydrogel.

Figure 4B presents the stress–strain curves of AHG and CAHG. After adding CAP into agarose, the fracture strain and fracture stress are weaker than in the original agarose hydrogel. It destroys the dense structure of agarose hydrogels and reduces their mechanical properties.

### 2.5. AIE Effect Verification of CAP

**Figure 5:**
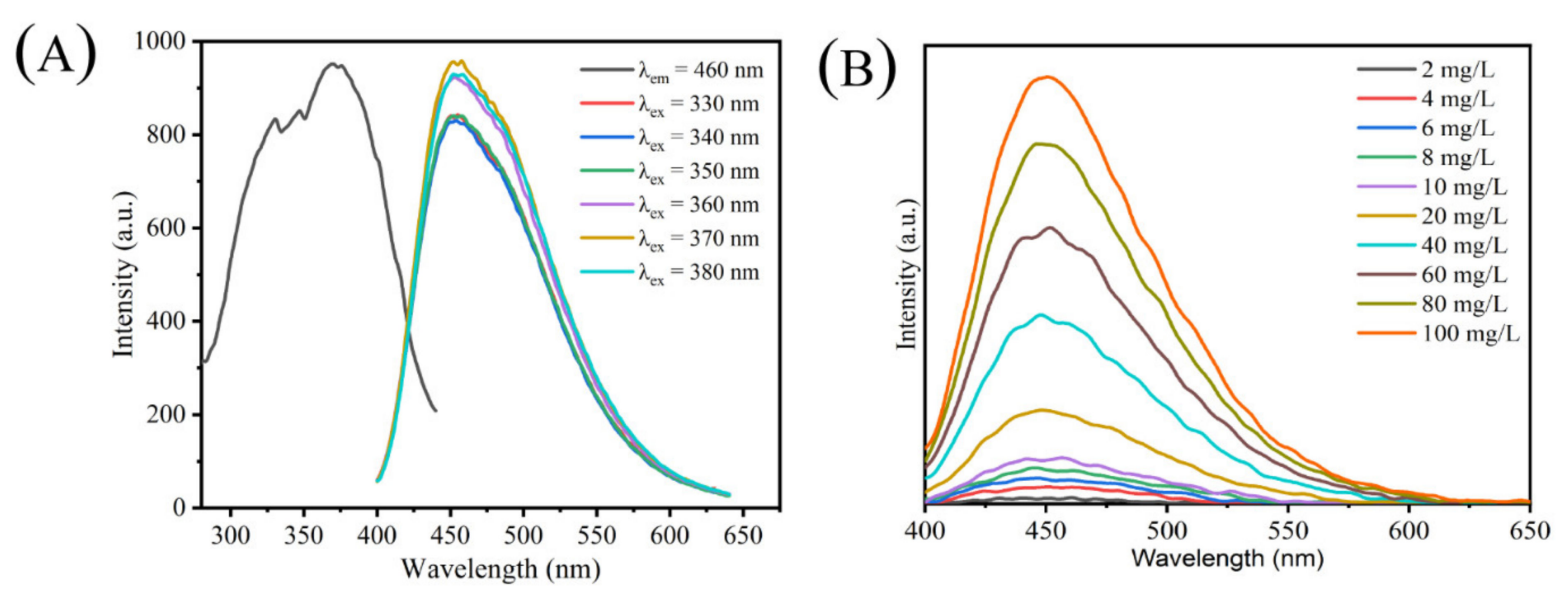
Fluorescence emission and excitation spectra of CAP (**A**); fluorescence emission spectra of CAP in water with different concentrations (**B**).

Figure 5A shows the excitation spectrum and emission spectrum of the fluorescence spectrum of CAP in the solid state, and the emission peak is at about 460 nm. The position of the fluorescence emission peak does not change with the position of the excitation light. The energy level gap does not change with the excitation, and the surface functional groups are stable. Figure 5B shows the fluorescence emission spectra of CAP aqueous solutions with different concentrations. With the increase in the concentration, the luminescence intensity of the solution increases gradually, which is a typical aggregation-induced luminescence phenomenon (AIE). With the increase in the molecular concentration, aggregation between molecules gradually occurs, and nonradiation channels such as molecular vibration or internal rotation are limited, which enhances the radiation channel and increases the fluorescence intensity [30,44].

### 2.6. Photophysical Properties of CAP

**Figure 6:**
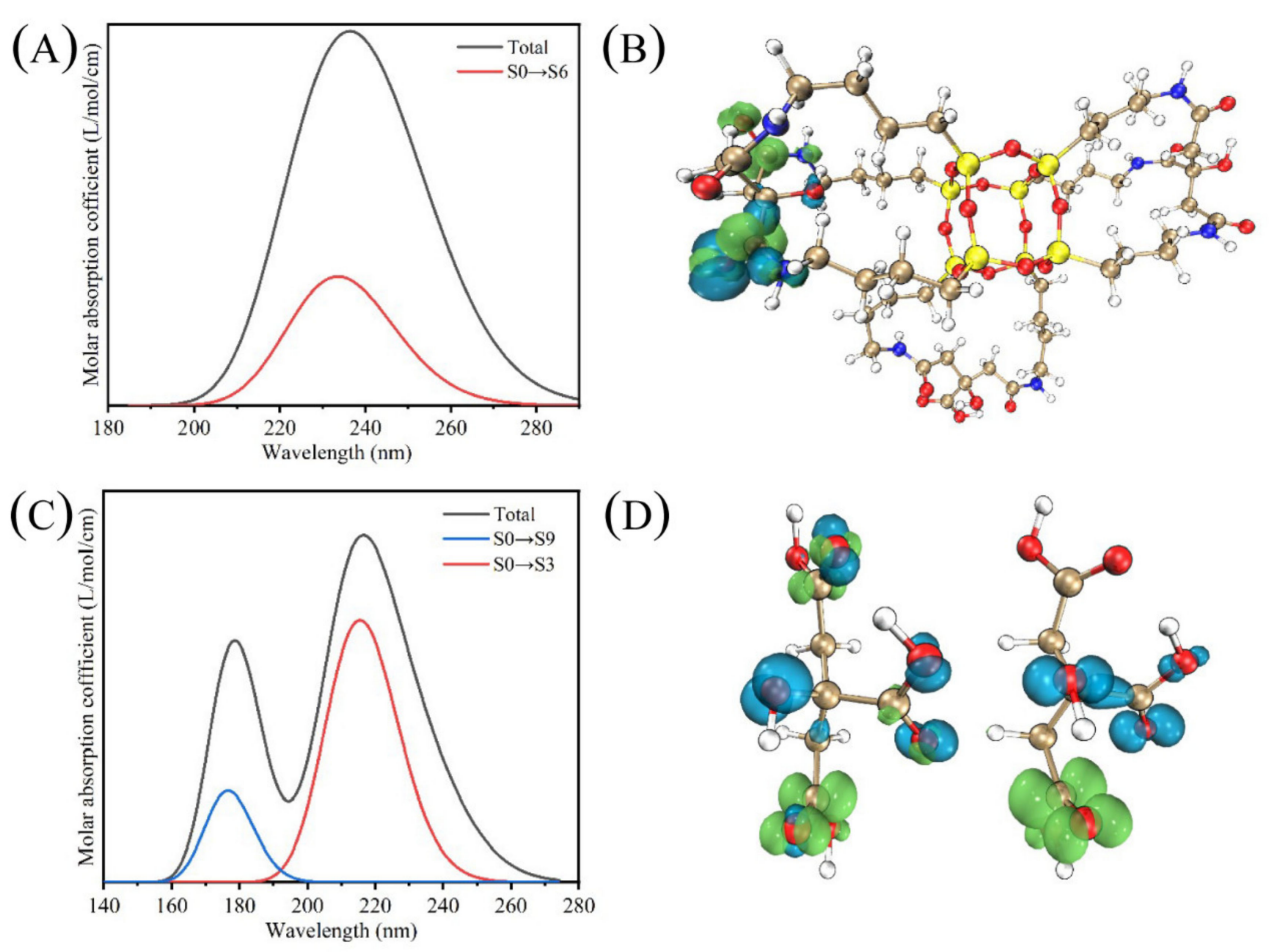
Simulated electronic absorption spectrum (**A**) and real space representation of hole and electron distribution for S0 → S6 of CAP (**B**); simulated electronic absorption spectrum (**C**) and real space representation of hole and electron distribution for S0 → S9 and S0 → S3 of CAP (**D**).

To obtain insights into the electron distribution of CAP, we employed the DFT and TD-DFT theory at the M062X-D3 (BJ)/6-311g(d) level to calculate electronic absorption spectrum of CAP by Gaussian analysis. As shown in Figure 6A, there is an absorption peak at 236.4 nm. The peak is mainly from four transitions (Table 2): 33.6% from S0 → S6, and the oscillator strength (f) is 0.0092; 17.4% from S0 → S2; 13.1% from S0 → S1 (f = 0.0056); and 10.3% from S0 → S8 (f = 0.0035). Therefore, the electron absorption of CAP mainly comes from S0 → S6. For this reason, the hole electron analysis was applied to the above S0 → S6 excitation.

It can be seen from the hole electron diagram of CAP (Figure 6B) that the holes and electrons excited by S0 → S6 are mainly concentrated in a part of the amide bond. Therefore, the excitation type belongs to the local excitation in the amide part. The holes are mainly distributed on both sides of the carbonyl oxygen, while the electrons are mainly located in the C=O. There are two cross-sections along or perpendicular to the bond axis. Therefore, the distribution of electrons is mainly composed of π* orbitals, and the electrons are mainly from the lone pair electrons of oxygen, so the S0 → S6 excitation type is n → π* local excitation.

Through the above discussion, it can be concluded that the silicon core of POSS hardly participates in excited state electron transfer. Hence, in order to further explore the optical mechanism of CAP, we used the same level of the TD-DFT theory above to calculate the electronic absorption spectrum of citric acid (Figure 6C). There are two strong absorption bands at 178.6 and 216.5 nm, which belong to S0 → S9 (f = 0.0029) and S0 → S3 (f = 0.0083) excitation, respectively.

In the hole electron diagram (Figure 6D), during the S0 → S9 transition of citric acid, the holes are mainly distributed on the oxygen of the hydroxyl and carboxyl groups connected by the middle carbon, and a small amount are distributed on the carbonyl oxygen at both ends. The excited electrons are mainly distributed in the carbonyl groups at both ends and have two cross-sections along or perpendicular to the bond axis. Therefore, the distribution of electrons is mainly composed of π* orbitals. The main part of the holes is principally located in the hydroxyl and carboxyl part connected by the central carbon, and the main part of the electrons is principally located in the carboxyl part at both ends. The electrons and holes have very high separation. Therefore, S0 → S9 is the n → π* charge transfer excitation from the hydroxyl and carboxyl group of the intermediate carbon to the carboxyl groups on both sides.

When the S0 → S3 transition occurs, the holes are mainly distributed in the hydroxyl oxygen and carboxyl oxygen on the central carbon, while the excited electrons are mainly distributed in the carbonyl part at one end. There are two cross-sections along the bond axis, or perpendicular to the bond axis. Thus, the electron distribution is mainly composed of π* orbitals, and the principal part of the electrons is located in the carboxyl part at one end. The principal part of the holes mainly exists in the carboxyl and hydroxyl groups connected by the central carbon. The electrons and holes have very high separation. Hence, S0 → S3 is the n → π* charge transfer excitation from the hydroxyl group and carboxyl group on the intermediate carbon to the carboxyl group on one side.

Although the core structure of POSS does not participate in electronic excitation, the rigid structure of POSS changes the excited state properties of the introduced citric acid, turning its original charge transfer excitation into local charge excitation.

### 2.7. Ion Detection

#### 2.7.1. Ion Selectivity and Fe^3+^ Adsorption

**Figure 7:**
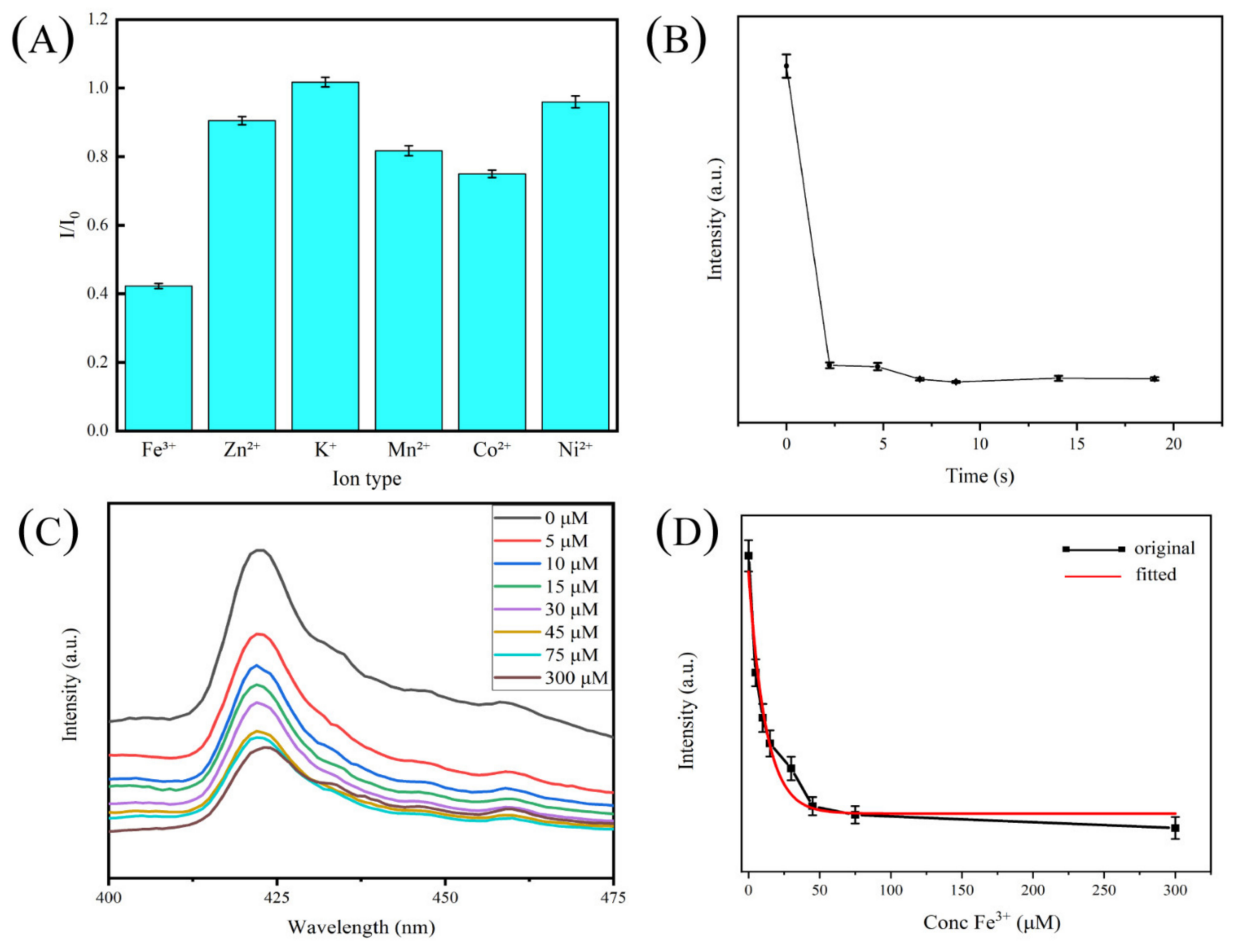
Effects of different ions on fluorescence intensity of CAHG (**A**); quenching effect of Fe^3+^ on CAHG at different time (**B**); fluorescence spectra of CAHG with the presence of increasing amount of Fe^3+^ anion (**C**); variation curve of fluorescence intensity with Fe^3+^ concentration (**D**).

Selectivity is the key parameter of a fluorescent probe, so we analyzed and compared the selectivity of CAHG to Fe^3+^. CAHG has a strong fluorescence response to Fe^3+^, but a weak fluorescence response to other ions. Figure 7A is a ratio diagram of fluorescence intensity after immersion of CAHG in an equal amount of metal ions (I) and blank solution (I_0_). It can be seen that only Fe^3+^ among many ions can cause a CAHG fluorescence-quenching response. This may be attributed to the coordination between amide groups in CAP and Fe^3+^, causing energy and electron transfer, leading to fluorescence quenching.

The response of CAHG to Fe^3+^ at different reaction times is shown in Figure 7B. When CAHG is added into 500 μM Fe^3+^, the fluorescence is almost quenched, and is unchanged after about 2.5 s. This shows that CAHG can be used for detecting Fe^3+^. As shown in Figure 7C, the fluorescence intensity at 425 nm is obviously quenched. When the concentration of Fe^3+^ is greater than 45 μM, the fluorescence intensity of the reaction system tends to be stable. The lowest Fe^3+^ concentration that CAHG can detect is 5 μmol/L. Figure 7D is the variation curve of fluorescence intensity with the Fe^3+^ concentration. The fitting equation is y = 283.84423 × exp(−x/11.31402) + 179.05944, while R^2^ is 0.97307. Through the equation, we can obtain the concentration of unknown Fe^3+^ within a certain concentration range by the fluorescence intensity change of CAHG. Thus, CAHG has the potential to be used as a fluorescence sensor.

#### 2.7.2. Ion Adsorption

Because agarose has a good adsorption ability, CAHG can adsorb objective ions in aqueous solution when used as fluorescent probe. The fluorescence intensity of CAHG decreases with the increase in the Fe^3+^ concentration, which is mainly due to the large amount of carbonyl and amide groups in CAP. Due to agarose having stronger adsorption properties, Fe^3+^ is gradually absorbed into the hydrogels and produces coordination effects with the carbonyl oxygen and amides in CAP. At this time, owing to the existence of coordination, most of the energy of CAP in the excited state is transmitted to the metal ions in a nonradiative way, which greatly reduces its own radiation channel. Therefore, the fluorescence emitted by CAP gradually weakens. In addition, since Fe^3+^ does not have photoelectric properties, the absorbed energy is consumed in nonradiation channels, and does not fluoresce.

In order to explore further applications of hydrogels, we used inductively coupled plasma (ICP) to explore CAHG’s adsorption of Fe^3+^ in solution. We placed CAHG in Fe^3+^ solution and stirred it for 12 h. Then, we removed the suspension in the mixture by centrifugation, and detected the residual content of Fe^3+^ in the solution. The removal efficiency of metal ions can be calculated by the following formula:

(1) $$r=\left(1\;-\;\frac{C_{1}V_{1}}{C_{0}V_{0}}\right)\;\times \;100\%$$

(2) $${\;q}_{t}=\frac{\left(C_{0}{\;-\;C}_{1}\right)V}{M}$$

where “r” represents the adsorption rate, C_1_ (M/mL) represents the residual concentration of Fe^3+^, C_0_ represents the initial concentration of Fe^3+^, V represents the volume of solution (V_1_ = V_0_), and M represents the quality of agarose in the hydrogels.

**Figure 8:**
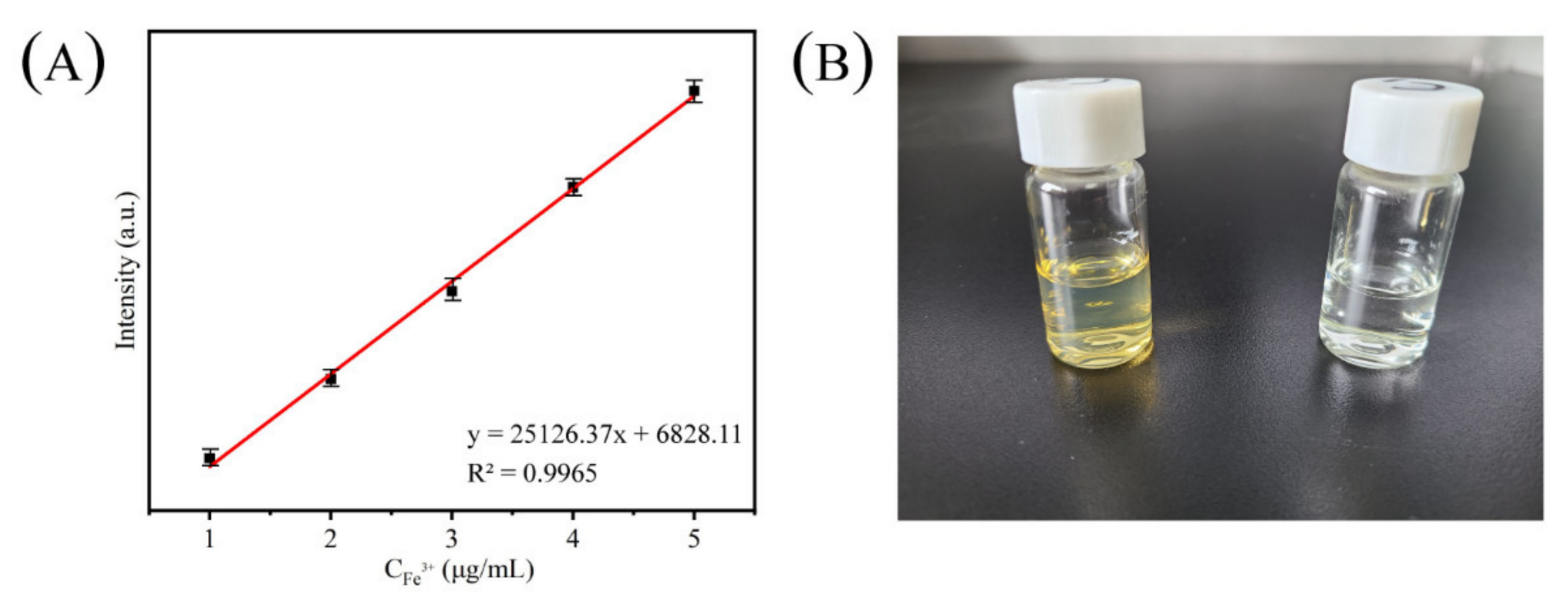
The standard curve for Fe^3+^ (emission intensity corresponding to metal ion content) (**A**); variation of solution before and after adsorption of Fe^3+^ by CAHG (**B**).

According to the experimental measurement results and then linear fitting (Figure 8), the regression equation of the standard curve is y = 6828.11 + 25,126.37x, and the square R^2^ of the correlation coefficient is 0.9965, while “y” represents the intensity of the iron ion emission peak and “x” represents the iron ion concentration. As shown in Table 3, the Fe^3+^ concentration before adsorption is 9.23 × 10^−6^ M, and the concentration after adsorption is 6.77 × 10^−6^ M. The adsorption rate of CAHG to Fe^3+^ is about 27%. The adsorption capacity is about 26.75 mg/g, which is higher than some other adsorption materials [45,46,47,48,49,50,51].

#### 2.7.3. Evaluation of Photothermal Properties of CAHG-Fe^3+^

**Figure 9:**
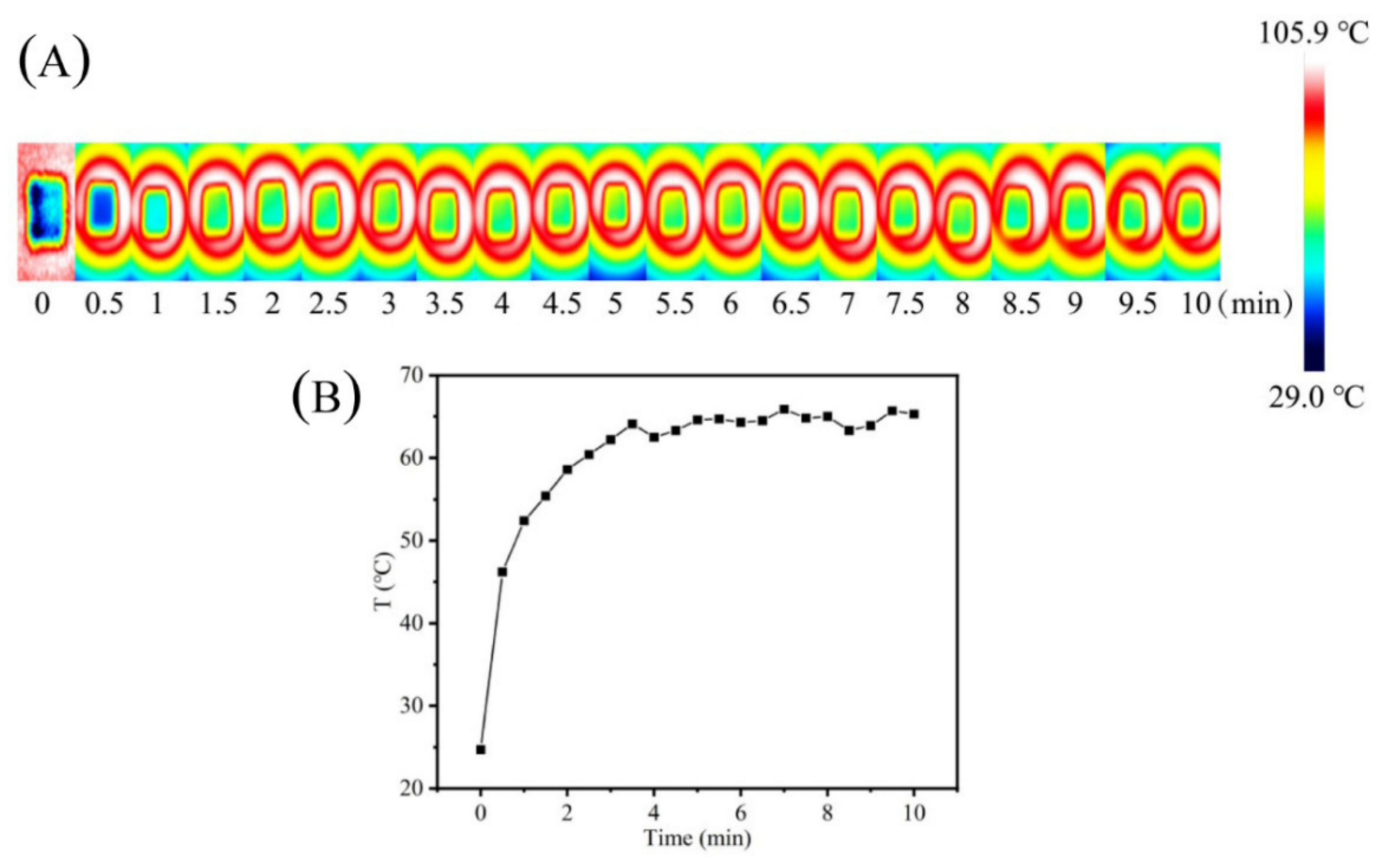
Infrared thermographic image of CAP agarose hydrogel under sunlight irradiation (0.65 W/cm^2^) (**A**) and temperature variation curve (**B**).

In order to evaluate the photothermal effect of CAHG after adsorbing Fe^3+^, we explored the photothermal effect of CAHG-Fe^3+^ at different exposure times with a solar light source of 0.65 w/cm^2^ and detected the temperature change by a thermal imager. As shown in Figure 9, with the increase in exposure time, the temperature of CAHG-Fe^3+^ continued to rise. After 4 min, the temperature reached the maximum of 65 °C, and the temperature remained basically unchanged from 4 min to 10 min. This shows that CAHG-Fe^3+^ can be further used as photothermal material, allowing for material recycling and reducing material waste.

## 3. Conclusions

In summary, we synthesized citric acid POSS (CAP) from citric acid and aminopropyl POSS (OAP-POSS) and added CAP into agarose to prepare CAP-agarose hydrogel (CAHG). The CAP showed excellent fluorescence properties and had rich active groups on the surface, such as carbonyl and amide groups. In addition, we also explored the luminescence mechanism of CAP through theoretical calculations. The prepared fluorescent hydrogel retained good fluorescence characteristics and fluorescence quenching of CAP, which could detect Fe^3+^ in solution, and showed a high sensitivity fluorescence response to Fe^3+^. The experimental results show that the lowest Fe^3+^ concentration that CAHG can detect is 5 μmol/L, and the adsorption capacity of Fe^3+^ is about 26.75 mg/g. Through the equation, we found the Fe^3+^ concentration (in a certain concentration range) by measuring the fluorescence intensity of CAHG, which showed that CAHG has the potential to be used as a fluorescence sensor. The prepared fluorescent hydrogel is a pollution-free and reused material. It has great potential in fluorescent probes, ion adsorption, and fluorescence sensors, and it can also be used as a photothermal material after adsorbing Fe^3+^.

## 4. Materials and Methods

3-Aminopropyltriethoxysilane (APTES), phthalic anhydride, citric acid and agarose, dimethyl sulfoxide, concentrated hydrochloric acid, anhydrous ethanol, methanol, tetrahydrofuran, and imidazole (AR, Macklin Corporation, Shanghai, China) were all used directly, without purification.

### 4.1. Preparation of POSS

The OAP (aminopropyl POSS) was synthesized according to a previous report [52]. We added APTES (7.5 mL) to CH_3_OH (180 mL) under magnetic stirring. After that, 15 mL HCl was added, and the mixture was refluxed for 19 h at 90 °C. After the reaction was cooled to room temperature, excess THF was added to the solution to obtain white precipitate OAP. Then, the crude product was obtained by vacuum filtration. Next, the solid was dissolved in a certain amount of deionized water. We used NaHCO_3_ to adjust the pH to neutral. Finally, the colorless and transparent solution was carefully transferred to a dialysis bag (interception Mn = 0.5 kDa) and dialyzed with distilled water for 24 h to remove impurities. The pure OAP solid was obtained by freeze-drying (Scheme 1A).

For the preparation of CAP(citric-acid-POSS), we placed citric acid (0.6 g) and OAP (4.118 g) in a beaker and added deionized water (15 mL). After stirring and dissolving, we put the clear and transparent mixed solution into a reactor, and reacted it at 160 °C for 4 h. After cooling, the solution was transferred to a dialysis bag (interception Mn = 0.5 kDa) and dialyzed with distilled water for 24 h to remove impurities. Solid CAP was obtained by freeze-drying (Scheme 1A).

### 4.2. Preparation of Agarose Hydrogel

For the preparation of AHG, we added agarose powder (1.8 g) to deionized water (150 mL) under magnetic stirring and controlled the temperature above 90 °C to melt it completely. After cooling, we divided the hydrogel into small pieces of the same size and weight.

For the preparation of CAHG, considering the fluorescence properties (Figure 5) and mechanical properties of hydrogels, a moderate amount of CAP was selected. We put 20 mL of CAP deionized water solution (30 μM or 40 mg/L) into agarose powder and water, and repeated the above experimental operation.

### 4.3. Characterization

The infrared spectra (FT-IR) of samples were obtained with a Fourier transform infrared spectrometer (PerkinElmer, Waltham, MA, USA). The MS of OAP and CAP were measured by matrix-assisted laser desorption ionization time-of-flight mass spectrometry (Autoflex III, Bruker, Karlsruhe, Germany). The structure of CAP was characterized by a transmission electron microscope (TEM) (JEM 2100F, JEOL, Tokyo, Japan). The micromorphology of AGs was characterized by a scanning electron microscope (SEM) (QUANTA, FEI, Eindhoven, The Netherlands). The thermal performance of AHGs were characterized by a thermogravimetric analyzer (TG209F3, Netzsch, Waldkraiburg, Germany). The fluorescence spectra (PL) were determined by a fluorescence spectrophotometer (LS55, PE, Cincinnati, OH, USA). The XPS was measured by an X-ray photoelectron spectrometer (K-Alpha, FEI, Eindhoven, The Netherlands).

### 4.4. Theoretical Calculation of CAP

The theoretical calculation mainly used Gaussian 09 and Multiwfn. The geometric optimization calculation of the S0 state was carried out based on the DFT [53,54] method b3lyp-d3/6-311G (d). TD-DFT [55,56] calculations were performed based on functional m062x-d3 (BJ) to obtain the electron absorption spectra. The distribution between holes and electrons was explored by using Multiwfn [57] software, based on hole electron theory.

## Figures and Tables

**Table 1:** Schedule of experiment instruments.

Sample	First Stage		Second Stage		Weight
	T (°C)	Weight Loss (%)	T (°C)	Weight Loss (%)	Remaining after 550 °C (%)
AHG	125.1	12.4	433.6	63.3	18.1
CAHG	142.6	10.8	470.5	59.3	26.2

**Table 2:** Excited state transition with TD-DFT for CAP.

Transitions	f	E (eV)	Contribution
S0 → S6	0.0092	5.3082	33.6280%
S0 → S2	0.0058	5.0560	17.3790%
S0 → S1	0.0056	4.9711	13.1280%
S0 → S8	0.0035	5.4415	10.3130%

**Table 3:** The ICP data of CAP hydrogel with Fe^3+^.

Ion	Fe^3+^
Initial concentration (M)	9.23 × 10^−^^6^
Residual concentration (M)	6.77 × 10^−^^6^
Adsorption rate (%)	27
qt (mg/g)	26.75

## Data Availability

Data are contained within the article.

## Supplementary Materials

The following are available online at https://www.mdpi.com/article/10.3390/gels7040173/s1, Figure S1: MALDI-TOF spectrum of (A) aminopropyl-POSS (OAP) and (B) citric-acid-POSS (CAP), Figure S2: SEM images with different resolutions of AG (A,C,E) and CAG (B,D,F), Table S1: Comparison of various substitutions between calculated and experiment values.
