# Supplementary material for: Preparation of Agarose Fluorescent Hydrogel Inserted by POSS and Its Application for the Identification and Adsorption of Fe3+
Source: Gels. 2021 Oct 18;7(4):173. doi: 10.3390/gels7040173 (PMC8544435; doi:10.3390/gels7040173)
Supplement: Supplementary file 1 [file gels-07-00173-s001.zip › gels-1387656-supplementary.pdf]

Supplementary materials

Preparation of Agarose Fluorescent Hydrogel Inserted by POSS and Application to Identification and Adsorption of Fe^{3+}

Zhengquan Fu [1, 2, 3, +], Ming Li[1, 2, 3, +], Yuanhang Li [1, 2, 3], Zhiyuan Zhang [1, 2, 3], Di Wang [1, 2, 3, *], Chengyu Wang [1, 2, 3], Jian Li [1, 2, 3]

1 Key Laboratory of Bio-based Material Science and Technology (Ministry of Education), Northeast Forestry University, Harbin 150040, P.R. China;
2 Engineering Research Center of Advanced Wooden Materials (Ministry of Education), Northeast Forestry University, Harbin 150040, P.R. China;
3 Collage of Material science & engineering, Northeast Forestry University, Harbin 150040, P.R. China.
fzq1999fzq@163.com (Z. F.); alexlavie@outlook.com (M. L.); lyh@nefu.edu.cn (Y. L.); blairzhang96@outlook. com (Z. Z.); diwang1030@nefu.edu.cn (D. W.); wangcy@nefu.edu.cn(C. W.); nefujianli@163.com (J. L.)
* Correspondence: diwang1030@nefu.edu.cn; Tel.: +86-1864-506-5081
\+ These authors contributed equally to the work

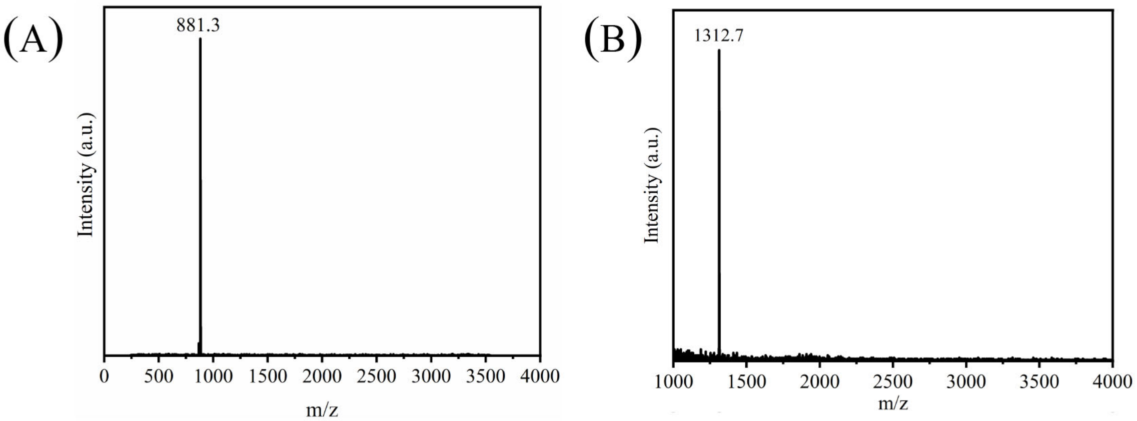

Figure. S1. MALDI-TOF spectrum of (A)aminopropyl-POSS (OAP) and (B) citric-acid-POSS (CAP)

Table S1. Comparison of various substitutions between calculated and experiment values

Formula Structure	Experimental Value (m/z)	Calculated Value (m/z)
$[(C_3H_8N)_8(Si_8O_{12})+H]^+$	881.3	882.51
$[C_{42}H_{72}N_8O_{25}Si_8+H]^+$	1312.7	1313.75

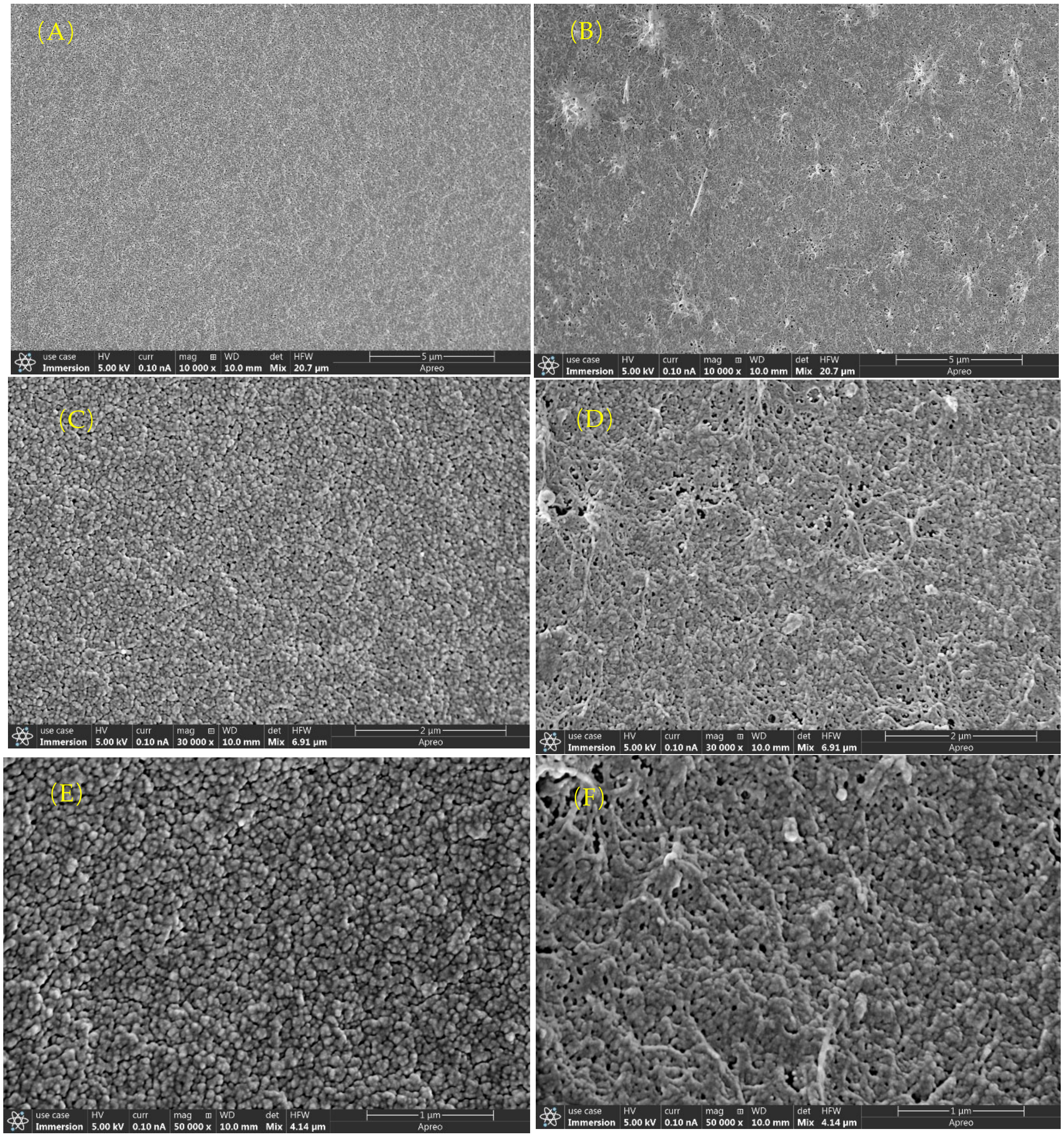

Figure. S2. SEM images with different resolutions of AG (A, C and E) and CAG (B, D, and F)
